# The role of partner support for health behaviours in people living with and beyond cancer: A qualitative study

**DOI:** 10.1002/pon.6032

**Published:** 2022-09-26

**Authors:** Natalie Gil, Abigail Fisher, Rebecca J. Beeken, Simon Pini, Natalie Miller, Caroline Buck, Phillippa Lally, Rana Conway

**Affiliations:** ^1^ Research Department of Behavioural Science and Health University College London London UK; ^2^ Leeds Institute of Health Sciences University of Leeds Leeds UK

**Keywords:** cancer, health behaviours, interdependence, living with and beyond cancer, oncology, partner support, qualitative, social support

## Abstract

**Objective:**

This study aimed to qualitatively explore how partner support for health behaviours is perceived, received, and utilised in people living with and beyond cancer (LWBC).

**Methods:**

Semi‐structured audio interviews were conducted with 24 participants, 15 men and nine women, living with and beyond breast, prostate, and colorectal cancer. Inductive and deductive Thematic Analysis was used to analyse the data.

**Results:**

Three key themes with six subthemes were identified relating to partner support for health behaviours: (1) Interdependence (Reciprocity, Overt Control, Influence & Motivation) (2) Concordance (Shared Attitudes & Health Beliefs, Shared Health Behaviour) and (3) Communal Coping (Communal Orientation towards Health and Decision Making, Co‐operative Action in Health Behaviour).

**Conclusions:**

Partner support plays a unique and significant role in the health behaviours of people LWBC. Partners play a collaborative role in managing health and facilitating health behaviours, while the high level of concordance in couples may represent a potential barrier to change via the reinforcement of maladaptive health beliefs and behaviours.

**Implications for Cancer Survivors:**

Overall, findings demonstrate that partners should be considered and included where possible when designing future behaviour change interventions for people LWBC.

## 1 BACKGROUND

Cancer incidence is increasing worldwide with a projected 23.6 million new cases per year by 2030.^1^ Advances in detection, early diagnosis and treatment mean there are now more people living with and beyond cancer (LWBC). Numbers are expected to increase by 3% annually, with an estimated 1 million survivors per decade by 2040.^2^ There is increasing recognition that tailored long‐term support, informed by chronic disease models of care, must include interventions encouraging lifestyle changes to promote health, well‐being, and survival.^3^

There is strong evidence that symptoms, Quality of Life (QoL), and survivorship can be significantly improved by targeting multiple health behaviours, including physical activity (PA), diet, smoking and alcohol consumption.^4^ Several meta‐analyses have found increased PA post‐diagnosis improved survival outcomes in 11 cancer types,^5^ reduced breast cancer deaths by 34%, and reduced all cause mortality by 41%.^6^ Furthermore, a higher intake of vegetables and fish was inversely associated with overall mortality, while a ‘Western’ dietary pattern was associated with overall mortality.^7^ The strongest evidence for the efficacy of behavioural interventions is for breast, prostate and colorectal cancer and based on this empirical evidence, the World Cancer Research Fund has developed guidance for health professionals to help improve health behaviours in people LWBC.^8^

Despite the benefits of adopting positive health behaviours, studies have shown that many people LWBC are not meeting health recommendations.^9^, ^10^ It is possible that clearer messaging and interventions from healthcare professionals could help improve health behaviours in this population and it has been suggested that a cancer diagnosis may present a ‘teachable moment’ whereby patients are open to making changes in lifestyle in response to a major health concern.^11^ Moreover, a body of empirical evidence suggests that while long‐term behavioural change can be difficult, it may be facilitated by concomitant support from the social environment^12^ and that individuals attempting to make behavioural changes can be positively influenced by their significant others during the course of this process.^13^

Social support is considered one of the major social influences on health behaviour.^14^ The smallest network is of course the dyad and using one‐to‐one peer matched support has proven effective in breast cancer survivors and their daughters.^15^ In addressing the impact of dyadic support on health behaviours of people LWBC, intimate partner relationships are of particular interest as partners have profound influence on one another, and health behaviour is often concordant across couples.^16^ Moreover, partner support has been found to improve outcomes across a range of domains including smoking^17^ and PA,^18^, ^19^ and improve cancer outcomes, lessen pain and lower mortality.^20^ While few post‐treatment interventions currently target cancer survivors and partners, positive findings from a recent scoping review indicate that there is potential for expanding this area of research^21^ and several feasibility studies have shown promising results with couples‐based behaviour change interventions including a PA intervention for breast and prostate cancer survivors, partnered strength training for prostate cancer survivors and a diet and exercise intervention for people living with and beyond breast, prostate and colorectal cancer.^22^, ^23^, ^24^ However, there remains relatively little qualitative research exploring how partner support is experienced for health behaviours by people LWBC. The aim of the present research was to qualitatively explore the role of partner support for people LWBC and how this support may influence and facilitate their health behaviours.

## 2 METHODS

### 2.1 Design

This was a qualitative study using one‐to‐one semi‐structured telephone interviews. The study adopted an interpretivist approach, suited to generating knowledge relevant for health and clinical practice.^25^ This approach recognises the importance of situating the researcher in the context of that which is being studied, in order that they may offer an interpretive understanding of the meaning participants attribute to their own experiences. This study was part of the Advancing Survivorship Cancer Outcomes Trial (ASCOT),^26^ a randomised controlled trial of a brief habit‐based health behaviour intervention for people living with and beyond breast, prostate, or colorectal cancer in the UK. Ethical approval was obtained through the National Research Ethics Service Committee South Central—Oxford B (reference number 14/SC/1369). An amendment was approved for a Covid‐19 follow‐up, in July 2020 which included a survey and qualitative interviews. Participants provided informed consent on paper at the start of the trial and online or over the phone for the Covid‐19 follow‐up. Methods and results are presented in line with Consolidated Criteria for Reporting Qualitative Research (COREQ) checklist.^27^

### 2.2 Recruitment and data collection

Participants who had received an initial diagnosis of breast, prostate, or colorectal cancer in 2012/2013 were recruited to ASCOT from 10 NHS Trusts across London and Essex between 2015 and 2019, randomised to receive a habit‐theory based behaviour change intervention or control, and then assessed at 0, 3, 6 and 24 months. Inclusion criteria for the trial were, adults (aged ≥18 years), diagnosed with non‐metastatic breast, prostate, or colorectal cancer, not currently receiving anti‐cancer treatment (except oral treatments taken at home), able to understand spoken and written English. Exclusion criteria included individuals receiving anti‐cancer treatment requiring hospitalisation, with metastatic cancer, or severe cognitive impairment. A follow‐up survey was completed in 2020–2021 to understand the impact of COVID‐19, where participants were given the option to consent to be contacted for qualitative interviews about factors influencing their health behaviours during the pandemic. Of the 788 survey respondents, 669 (85%) consented to interview, of which 573 indicated that they were married/living with partner. For the current study, participants were purposively sampled to ensure adequate representation of the three cancer types, gender, ASCOT intervention/controls, and rural/urban dwelling. It was important to hear the experiences of those who had received our behavioural intervention and those who had not, as well as participants living in both rural and urban areas, as location and access to commercial facilities and green spaces can have significant impact on health behaviour. Only participants who indicated they had a partner in the Covid‐19 survey were invited for interview. The sample size range was 15–25 participants, with data gathering to stop when thematic saturation was reached. This range was deemed adequately broad for one interviewer‐one participant research, while maintaining the capacity to provide richly textured information.^28^ Partner status was reconfirmed at the beginning of each interview. A topic guide was developed (see Supporting Information S1) covering areas of interest in relation to diet, PA, smoking and alcohol, with prompts to guide conversations to how partner support is perceived, received, and utilised in the relation to these domain. Each topic was explored sequentially and in‐depth, giving participants every opportunity to reflect on, describe, and detail their experiences. One‐to‐one interviews were conducted via telephone by a female Health Psychology Researcher (NG) with no prior relationship with any participants. Comprehensive notes were taken during and immediately following each interview. Interviews were audio recorded and transcribed verbatim by a professional transcription company.

### 2.3 Analysis

Initially, 15 interviews were conducted by NG, audio files were listened to twice to become familiar with broad themes of conversations and to facilitate early identification of patterns, before being coded and analysed using Thematic Analysis, with extensive referral to methods employed by Braun and Clarke.^29^ Six stages of familiarisation, initial code generation, searching for themes, reviewing themes, defining themes, and writing up were followed. Analysis of the transcripts was informed by inductive methods to derive themes from the data and deductive methods to situate those findings within a theoretical model. To reduce single method, single‐researcher bias, three transcripts were second‐coded independently by RC, while emerging ideas were regularly discussed with the research team. Once an initial coding framework was agreed upon, the remaining interviews were conducted in sets of three until thematic saturation was reached. Inductively derived codes were mapped to central constructs of Social Support theory^30^ Emotional, Tangible, Informational and Appraisal support.^31^, ^32^ Interdependence theory^33^ and Theory of Communal Coping^34^ were introduced to support the analysis. Data was managed using an Excel spreadsheet and organised methodically within a participant‐led theme matrix to ensure clarity, to avoid losing context and tone of conversations, and to demonstrate clear justification of the pathway from coding to conclusions drawn. The process was iterative, and codes and themes were continuously adapted to ensure data was accurately reflected in the findings. Illustrative quotes are provided stating participants gender, age, and cancer type.

## 3 RESULTS

In total, 15 (62.5%) men and 9 (37.5%) women were interviewed, including seven participants with a diagnosis of prostate cancer, seven with breast cancer and seven with bowel cancer. One participant had prostate and colorectal cancer, one had prostate and skin cancer and one had breast and anal cancer. See Table 1 for participant characteristics.

**TABLE 1:** Sample characteristics of interview participants

	Mean (range)
Age	66 (53–86)
**Gender**	*n* (%)
Male	15 (62.5)
Female	9 (37.5)
**Ethnicity**	*n* (%)
White British	22 (91.66)
White other	1 (4.16)
Indian	1 (4.16)
**Rural/Urban dwelling**	*n* (%)
Village/small town	9 (37.5)
Large town/city	15 (62.5)
**Living situation**	*n* (%)
With spouse	16 (66.66)
With spouse and immediate family/children	8 (33.33)
**Cancer type**	*n* (%)
Breast	7 (29.2)
Prostate	7 (29.2)
Colorectal	7 (29.2)
Breast and anal	1 (4.16)
Prostate and colorectal	1 (4.16)
Prostate and skin	1 (4.16)

**FIGURE 1:**
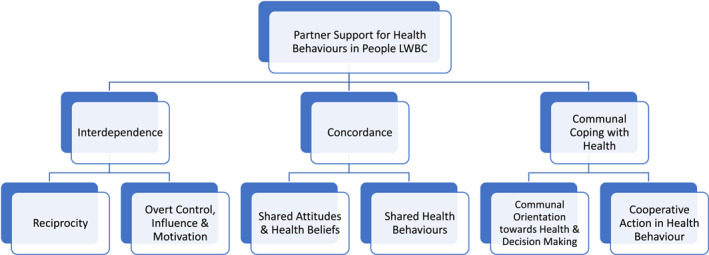
Partner support for health behaviours in people living with and beyond cancer: thematic map

Participants ranged in age from 53 to 85 years, and all were married, with the length of marriage ranging from 26 to 64 years. There were nine living in a village/small town, while 15 lived in a large town/city. Fourteen participants had been assigned to the ASCOT intervention group, 10 were in the control (usual care) group. Interviews were between 30 and 90 min long. Three themes with six sub‐themes were identified in the analysis, summarised in Figure 1, and discussed below.

### 3.1 Interdependence

Understanding the role of partner support for health behaviours revealed the importance of these dynamics in the everyday lives of people LWBC. ‘We’ was the pronoun of choice for participants when discussing their health behaviour. This early observation set the scene for the overarching theme of interdependence that was identified. This was evident in all relationships and took differing forms depending upon the existing dynamics of the relationship.

#### 3.1.1 Reciprocity

Participants referenced reciprocal support to their spouse and there was a strong sense of caregiving partnerships throughout discussions. Notably, participants duty to their spouse and their positive experience of life appeared to remain, even when their own abilities were diminished following their cancer diagnosis. One participant mentioned he was ‘*considering buying a scooter to keep up*’ (Male, 79, Prostate and Skin Cancer) to enable them to continue walking together. Many participants discussed the proactive role their partners play in facilitating PA, one expressed that having his partner with them helped to ease his feelings of vulnerability while out on his daily walk.

Sometimes I do go by myself but very rarely, not a good idea because I could be miles away from home, in the middle of a field somewhere, the only way they'd find me is through my mobile phone signal, so my wife comes with me

Male, 75, Colorectal Cancer

Multi‐morbidities were common amongst participants and their partners, and they described myriad ways in which they support each other with health issues. There was frequent reference to the heightened awareness partners felt they had of one another's health, and how they felt this placed them in a unique position to detect health changes that require attention.

They told me straight away I had Parkinson's. It took me completely by surprise. I thought, how am I going to tell my wife? So, I did tell her, and she said, “Yes, I thought so.”

Male, 73, Colorectal Cancer

Participants often discussed the important role their partners play in caregiving, or that their spouse had set up a lot of things for them following their cancer diagnosis, to make essential activities easier, such as showering. One participant discussed how their partner had adapted the home environment with ‘*a walk‐in shower, things like that for me to manage’* (Female, 59, Colorectal Cancer) or taken over certain activities such as shopping, that had become too difficult for them. There was a sense that partners ‘managed’ health and were ‘in charge’ of care. One participant referred to his wife as his ‘chief nurse’, while many discussed the high level of intimacy and emotional support involved.

She was in the next room. She would hear that alarm, she'd come in, she'd sort me out, sort the machine out, if the bag needed emptying, she would empty the bag. And she done that every night

Male, 74, Colorectal & Prostate Cancer

However, one participant directly linked her own breast cancer diagnosis with the stresses of managing her husband's ill health, describing the strain that taking over as the ‘provider’ for the family had taken on her, as well as making difficult decisions about raising her children without the active input and support of her husband. She described this period as a ‘*hard life*’ where she ‘*had to be the strong one*’. When discussing her breast cancer diagnosis, she expressed that during this period her health issues were ‘*non‐stop*’ and that she had her gallbladder removed, was diagnosed with hiatus hernia, and then with breast cancer, which she felt ‘*was all the stress I had gone through, it really affected my body*’ (Female, 57, Breast Cancer).

#### 3.1.2 Overt control, influence, and motivation

There were many instances of partners, particularly wives, exerting overt control and influence on health, with participants referring to their spouse as ‘the boss’ or the ‘decision maker of the house’ with respect to dietary behaviour. Many described the impact this had on their food choices, how it had changed their eating habits ‘*now I actually enjoy cabbage, runner beans*’ and ‘*it's that way she's influenced my food*’ (Male, 61, Colorectal Cancer). Participants welcomed depending on their partner and putting their trust in them to ‘make sure we do all the right things’. This appeared to provide a sense of security, stating ‘the encouragement helps’, although in many instances it is unclear from their statements how healthy their diet is.

Many participants described their partner as an important source of motivation for engaging in health protective behaviour, that they are proactive and ‘keep them on their toes’ particularly in relation to engaging in PA. That they ‘*push me to remain active*’ and would never ‘*let me sit back and have a duvet day*’ (Male, 78, Prostate Cancer). Others expressed how their companionship helped to support and encourage PA participation.

Togetherness I suppose, you know, whilst I'm having my PT [Personal Training] and moaning about something, she takes the piss out of me and vice versa, so it works.

Male, 55, Breast Cancer

In rare instances where couples chose to exercise separately, there was consensus that their partners provided positive support for this by facilitating the time spent on this activity, with participants noting that their partner ‘*didn't say you're never here to do the dishes because you're always out walking*’ (Male, 66, Prostate Cancer). One participant discussed how their partner feels exercising together is reassuring and by doing so they are able to monitor each other's overall health describing it as a benchmark of being able to assess each other's fitness.

Overall, participants appeared to value the frank appraisals partners offer of their health behaviour, often indicating their unique position of trust allows for a different level of input than one would tolerate from other sources.

It's someone you can accept it from, you can take it from your nearest and dearest. Even if it was a friend, you know, “Oh you're eating a lot.” You think, “mind your own business!” wouldn’t you? But if it's your partner then…

Female, 52, Breast Cancer

### 3.2 Concordance

Partners described a high level of concordance in their attitudes towards health and in their health beliefs and behaviours. Attitudes and health beliefs, both positive and negative, appeared to be reinforced by this consistency, with participants expressing the sentiment that there's no difference between themselves and their partners, and after so many years together, they have patterns and routines that they rarely deviate from, especially in relation to diet and exercise.

#### 3.2.1 Shared attitudes and health beliefs

During discussions about attitudes towards health and behaviours, many participants referred to their shared outlook, stating that ‘*we're both carrying a bit too much weight, but if we're exercising and keeping our arteries clear and not clogged, at least we're not going to die of a heart attack*’ (Male, 55, Breast Cancer). While others referenced their belief that they have the best possible diet that one can have.

There were two distinct responses to questions about health advice. Some participants felt themselves and their spouses were very receptive to such interventions and that ‘*my wife agreed with me that I should take part in as much of those sorts of things as possible*’ that ‘*if something does go wrong with,e there's more chance it will be spotted*’ (Male, 73, Colorectal Cancer). Others felt advice would be unnecessary and unwanted by both such as ‘*she would say we don't need it; we know what we are supposed to be doing*’ (Male, 74, Prostate Cancer), or ‘*people giving us advice? No, no, no*.’ (Male, 55, Breast Cancer). In some instances, agreement between spouses seemed impenetrable, with participants making bold statements that ‘*haven't been influenced by any recommendation; we make our own decisions*’ and that their behaviour ‘*cannot be improved*’ (Male, 72, Colorectal Cancer).

#### 3.2.2 Shared health behaviour

Most participants indicated high levels of behavioural concordance within their relationships, stating ‘*at our stage of life we have a pattern that suits us*’ (Male, 79, Prostate and Skin Cancer). Participants referenced their shared tastes in food and that ‘*We've always eaten tonnes of vegetables and fruit, probably more than most people really*’ (Male, 79, Prostate Cancer). There were many examples where participants referred to their daily walk together or shared love of bike rides. Others discussed how they had noticed a shared decline in activities noting since Covid they have done less. One participant mentioned ‘*we used to walk a lot but just recently she's got something wrong with her hip so we're having problems walking at the moment*’ (Male, 73, Prostate Cancer). This concordance was particularly evident in relation to alcohol consumption, there was a strong sense that partners don't engage in alcohol consumption without each other.

We would go out Friday and Saturday night, get up the next day, you've got a banging headache you've drunk too much, we'd both say never again. Then you eat Chinese, hangover food. Binge, then we'd be good all week. We're very similar, both of us have got no self‐control when it comes to alcohol

Female, 51, Breast Cancer

Participants appeared to have habitual behaviours with alcohol which remained constant across the life course. In couples who drink, this was seen as something to be enjoyed together or not at all. One participant explained that her husband had ‘*gone off drink since my diagnosis*’. She explicitly stated that, ‘*I don't want to drink on my own, so it's not very often*’ (Female, 65, Breast and Anal Cancer).

There were few instances of direct health behaviour discordance. One example was a couple where one was a smoker. She spoke of her husband's lifelong attempts to get her to stop stating ‘*he's been on at me for years to pack up smoking, says it'll kill you*’ (Female, 63, Breast Cancer). Despite this obvious difference, this was a couple with multiple morbidities. Overall, it was rare to hear of partners adopting a behaviour change individually. When one participant described changing an existing norm by giving up alcohol, it appeared to be an unwelcome challenge to the relationship and received little support.

It wasn't popular, to be honest. I think because it's something I'd just done unilaterally by myself

Male, 73, Colorectal Cancer

### 3.3 Communal coping with health

All participants described a collaborative approach towards their health and health behaviour. Partner's health was considered a joint concern, with overt expressions of communal coping. Participants stated that they had managed to cope with most things together, pulled together and managed to get through. They frequently detailed the practical steps taken to manage and facilitate their health behaviours.

#### 3.3.1 Communal orientation towards health and decision making

It was clear that partners viewed each other's health problems as ‘ours’ and partners took an active role in researching conditions and how to best manage them. One participant discussed his diagnosis, the theory that gut health is important, and how his wife ‘*investigated which probiotics were being used in trials and made sure I had a supply of those*’ (Male, 73, Colorectal Cancer). Many participants described their partners being much more vigilant over their health than they are themselves, especially in relation to Covid‐19, ‘*she was afraid I would get the damn virus, she was very strict, mask, hat, goggles, you name it*’ (Male, 72, Colorectal Cancer) and ‘*I was high risk, so my wife said I'd much prefer if you stayed at home*’ (Male, 55, Breast Cancer).

Participants discussed the large role their partner's support plays in medication adherence and attending check‐ups, how she will ‘*chase me up if I don't make my appointments, she's good on things like that*’ (Male, 73, Prostate Cancer) and how she ‘*encouraged me to do everything the doctor said and take part in all the studies*’ (Male, 73, Colorectal Cancer) to ensure they get the maximum support. In several instances, participants described placing trust in their partner to manage communication with healthcare professionals entirely, ‘*like with my doctors, they ring him because I didn't know anything about it.*’ (Female, 63, Breast Cancer).

This collaborative approach extends to information sharing and active involvement with medical decision‐making. Participants described their partners as having equal input regarding their treatment and care, that ‘*She was there at every consultation. She had an input and an opinion.*’ (Male, 75, Colorectal Cancer). There was a sense that participants place their trust in their partners to manage their health and health behaviour, that partners are ‘*always there and being very careful*’ (Male, 61, Colorectal Cancer).

#### 3.3.2 Cooperative action in health behaviour

Participants described frequent communication with their partner, and how they strategized to minimise negative impact of health problems. Some described how they ‘*did things by trial and error*’ (Male 75, Colorectal Cancer) together or made a conscious effort to change their diet together in response to a cancer diagnosis ‘*so, when I was doing the fasting, she does that as well*’ (Male, 63, Colorectal Cancer). Participants commonly discussed adopting new health protective behaviour with their partner in response to illness.

In instances where illness meant changing capabilities, it was clear that couples' function and respond as a unit, which adapts to make dyadic adjustments to accommodate these needs. It was notable that one partners physical limitations appeared to impact upon the other's exercise behaviour, for example, ‘*she suffers badly with the cold weather and the wind, we don't go out in bad weather*’ (Male, 73, Colorectal Cancer). Finally, participants appeared to feel comfortable with this high level of input from their partners in relation to their health. They consistently expressed depending upon their partners to ‘cope’ together.

My wife‐ She's a winner. I couldn't live without her. I don't like saying this sort of thing, but the day she passes away I’m going as well. I’m not going to be here without her, and I really mean that.

*Male, 77, Prostate Cance*r

## 4 DISCUSSION

This study found that partner support plays a unique and significant role in the health behaviours of people LWBC and extends understanding of the mechanism through which this influence occurs, the interdependent structure of the relationship. Three overarching themes of Interdependence, Concordance, and Communal Coping were identified. Interdependence appears to replace individualist paradigms of behavioural motivation (health beliefs, social support perception, self‐efficacy) with relational motivation, where health events are ascribed as meaningful for the dyad rather than simply for oneself. Concordance, (attitudes, beliefs, behaviour) and Communal Coping reflect this interdependence in action.

Within the present study, it was notable that participants were in highly compatible and supportive relationships and direct partner effects on health behaviours could be seen often, via partners overt control and influence. The findings suggest that partners are not only a ‘source’ of support but are actively engaged in coping with all aspects of each other's health, in a reciprocal and collaborative process. Interdependence theory further separates processes of influence into ‘joint’ and ‘mutual joint’ effects.^35^ The present data provides clear indication of joint effects, where the actions of the self and the partner impact upon the health of the individual. But perhaps most significantly, there was considerable evidence of mutual joint effects. That is, partner support was associated with partners engaging in health protective behaviours together, and in some cases initiating behaviour change for both in response to the within‐couple health threat of a cancer diagnosis. This suggests including partners in behaviour change interventions may be advantageous and supports recent preliminary findings that a couples‐based approach is significantly more efficacious in encouraging behaviour change, including physical activity, fruit and vegetable consumption and sustained weight loss among people LWBC, when compared to a survivor only programme.^22^

Reciprocity is a hallmark of supportive relationships and this study found repeated reference to reciprocal spousal support, lending further credence to the notion that partners are no longer self‐focused even after receiving a cancer diagnosis. The identity as partner, and focus on the roles and responsibilities this implies, continue to take precedence. Recent research^36^ suggests support provision is strongly associated with positive affect and health benefits in providers. However, as partners are often considered the primary source of support for many people LWBC, the challenges that this can present over time can also be detrimental to relationships. Research exploring perspectives of partners of those LWBC found the burden of cancer to be a contributing factor to relationship dissolution in over 50% of cases where separation occurred.^37^ In our study, participants were married for 25–50 years, indicating high levels of compatibility. In younger couples or in relationships where coping strategies are not shared, research has shown higher levels of psychosocial distress following a cancer diagnosis,^38^ which negatively correlates to quality of life and cancer‐related mortality.^39^

Our results are in line with previous findings which suggest that attitudes and health beliefs are highly concordant across couples.^16^ While some of this may be attributable to assortative mating, previous longitudinal research suggests that spousal influence itself is associated with health enhancing behaviours.^40^ The concept of ‘social contagion’ within couples is well documented across behaviours^41^ and is supported by studies attempting to change one partners risk behaviours, resulting in ‘behavioural diffusion’ which positively benefits the non‐participating partner.^35^ In a large‐scale study examining the influence of marital status on attendance at colorectal cancer screening, it was shown that married adults were more likely to attend screening than non‐married, and that inviting both members of a couple together further increased screening uptake.^42^ It is thought that partners monitor and regulate each other's behaviour in ways that influence health behaviour by means of ‘social control’.^43^ However, partners can also reach concordance through mutual reinforcement of unhealthy behaviours,^44^ as demonstrated in our study in relation to episodic binge eating and drinking.

From the outset of analysis, it was notable that ‘We’ was the first‐person pronoun of choice for all participants when answering questions about their health. Previous research which analyses We/I ratio scores, found a higher score was significantly associated with relationship quality and predicted positive changes in heart failure symptoms at follow up.^45^ Moreover, a study examining how couples describe coping with breast cancer found that resilient couples co‐ordinate their coping efforts by defining the cancer experience as a dyadic stressor, or ‘we‐stress’, that affects both, and this is evident in the ‘we‐language’ surrounding the descriptions of their experiences.^46^ Reflected in ‘We‐talk’ is another important construct of interdependence, Transformation of Motivation.^35^ This may explain how patterns of interdependence transpire and is a process by which couples move from self‐centred to relationship‐centred motivation for behaviour. Research suggests that Transformation of Motivation activates a communal approach to coping. Communal Coping^34^ draws a sharp distinction from social support, where resources are provided *from* one *to* another and is distinguished by its shared appraisals and sharing of resources.^47^ In the present study communal coping was demonstrated via participants clear appraisal of the health of one partner as a matter of mutual concern. Participants spoke of their in‐depth communication with their partners regarding health behaviours and described communal medical decision‐making and a cooperative approach towards actioning health protective behaviours, in many cases explaining that their partners co‐ordinated or ‘managed’ the response to illness.

### 4.1 Implications for research and clinical practice

Due to shared history and lifestyle, partners can provide intuitive and responsive support on a consistent needs‐be basis. However, it was notable that male participants mentioned placing complete trust in their partner to ‘*make sure we do all the right things*’ with regards to diet. This highlights the necessity to include partners from the outset when designing interventions to change dietary behaviour. In this study, participants appeared to rely upon the unique structural properties of their relationships to support health behaviours, and there was substantial between‐couple variation, while previous research suggests accounting for between‐couple differences when designing PA interventions for patients with osteoarthritis was acceptable and enhanced the support processes that help patients with osteoarthritis live healthier lives. This would indicate that development of a couple ‘health typology’ could be an excellent way to tailor future interventions to existing dynamics. While concordance may be positive in couples who partake in health protective behaviours, it is clearly a possible barrier to health in those who do not. Concordance of attitudes may also reinforce existing maladaptive health beliefs and behaviour. This further highlights the need to include partners in interventions to successfully challenge such health beliefs and change maladaptive within‐couple behaviours. While previous research suggests that it is strong communication and mutual trust that predisposes partners towards interdependence,^48^ it would be interesting to discover the extent of this transformation in younger couples and to explore the possibility of enhancing relationship‐centred motivations as part of future behaviour change interventions, which has shown some success in dyadic intervention studies.^49^

### 4.2 Study limitations

To our knowledge this is the first study to qualitatively explore the role of partner support for health behaviours in people LWBC and was conducted during the Covid‐19 pandemic, when participants were primed to consider their health behaviour closely. We recruited a majority male sample, where a breadth of experiences of LWBC were explored, including a male breast cancer survivor. There were several limitations. In research of this nature, there is both self‐selection and survival bias to consider. Our participants were heterosexual, of older age, and in marriages ranging from 26 to 64 years in length. Further research with same sex couples, individuals of varying sexuality and cohabiting individuals, or those who have been married for a shorter time, is needed to build upon our understanding of the implications of intimate partner relationships and their influence on health. A persistent problem with research of this nature is the concept of pseudo‐unilaterality^35^ which refers to bias that stems from continually examining one side of a two‐sided phenomenon. In the present interviews there were instances of background interjections where partners contradicted information being relayed and a new ‘mutual truth’ then presented. Ideally, future research should include independent interviews of both partners and a joint conversation, to fully understand the support needs of both partners and to establish where perspectives truly meet and where they diverge.

## 5 CONCLUSION

The study offers unique insights into how people manage health within couples and provides support for the development and utilisation of dyadic theory‐based behaviour change interventions for people LWBC. The study highlights the significance of the collaborative role of partner support for health behaviours in people LWBC and emphasises the interdependent nature of the human condition, especially in relation to health. Overall, partners represent important collaborators in behaviour change for people LWBC, which may be leveraged to great effect in future interventions.

## AUTHOR CONTRIBUTIONS

Natalie Gil, Rana Conway, Rebecca J. Beeken, Abigail Fisher, Natalie Miller, Phillippa Lally, Simon Pini, Caroline Buck designed the study. Natalie Gil designed the topic guide with revisions by Rana Conway, Abigail Fisher, Simon Pini and Caroline Buck. Natalie Gil conducted the interviews, analysed, and interpreted the data. Rana Conway independently coded three interview transcripts. Natalie Gil drafted and revised the manuscript, Rana Conway, Abigail Fisher, Rebecca J. Beeken, Simon Pini, Phillippa Lally, Caroline Buck, and Natalie Miller revised it critically and provided final approval for submitted version.

## CONFLICT OF INTEREST

The authors have no conflicts of interest to declare that are relevant to the content of this article.

## ETHICS APPROVAL

Ethical approval was obtained through the National Research Ethics Service Committee South Central—Oxford B (reference number 14/SC/1369) in January 2015. An amendment to incorporate an additional follow‐up to assess the impact of Covid‐19 using both questionnaires and qualitative interviews was approved in July 2020, and a further amendment to increase the number of qualitative interviews planned was approved in November 2020.

## CONSENT TO PARTICIPATE AND CONSENT FOR PUBLICATION

Informed consent was obtained from all individual participants included in the study.

## Supporting information

Supporting Information S1

## Data Availability

The datasets generated during and/or analysed during the current study are available from the corresponding author on reasonable request.
