# Supplementary material for: The role of partner support for health behaviours in people living with and beyond cancer: A qualitative study
Source: Psychooncology. 2022 Sep 26;31(11):1997–2006. doi: 10.1002/pon.6032 (PMC9828063; doi:10.1002/pon.6032)
Supplement: Supplementary file 1 — Supporting Information S1 [file PON-31-1997-s001.docx]

**Supplementary material**

**Interview schedule**

**Introduction**

- *Introduce self*
- *Thank them for taking part in this interview.*
- *Ensure anonymity*
- *There are no right or wrong answers, I’m just really interested in hearing your thoughts on the questions and I would particularly like to explore the role of partner support in health behaviours during the pandemic.*
- *Ask to begin recording*
- *We will begin with some general questions if that is ok with you.*

**Daily Life**

**Tell me about how you are spending your days at the moment?**

(*Are you working? From home? How about your partner, are they at home too?)*

*Has this changed since the covid-19 pandemic?*

**Home-life**

**Tell me about your current living arrangements**

*(Who’s at home?)*

Has this changed since the covid-19 pandemic?

**Home and Area**

**Can you tell me a little bit about your home and where you live?**

*(House or flat? Do you have a garden or outdoor space? Do you live near shops, supermarkets, parks, leisure centres? Do you live close to family and friends?)*

**Relationship**

**Can you tell me how long you and your wife have been married?**

*(Married? Live together? Children?)*

***Can you tell me a bit about your wife? How have you been getting along together during the pandemic? Is this usual?***

**I’m quite interested in hearing about your wife/husband’s role in your health behaviours.** Let’s start with food…

**Diet**

**How would you describe what you currently eat?**

**Has this changed since the pandemic?**

*(How many meals?*

*Do you follow a certain diet?*

*Who does the food planning and shopping?*

*How about preparing meals?*

*Do you cook for yourself? For your partner? How do you feel this helps you?*

*Do you eat together?*

*Do you like the same food?*

*What does your partner think about the foods that you eat?*

*Do you feel your partner has an influence on what you eat? How so?)*

**Have you made any changes in terms of food since your diagnosis?**

*(If so, what prompted this? Advice from a doctor or health professional? Family? How about in the past? Any changes at all? Any diets you may have tried? Weight watchers, slimming world etc? New Year resolution? How did it go?)*

**How about your partner?**

*(Are they aware you were making changes? How did that conversation go? Did they make changes too? How successful were you? Both? Together? Would you have liked more support from your partner? Or more involvement? Or Less involvement? Do you think them making changes would have helped you?)*

**Is there anything else you would like to cover about food before we move on?**

**Alcohol**

**Can you tell me a bit about your current alcohol consumption? Has this changed at all since the pandemic? Since Diagnosis?**

*(Do you drink at all? How often? Who with? How many drinks would you say you have in a week? Have you ever tried to make any changes to this? Why? How did your partner feel about these changes?)*

**How about your partner?**

*(Do they drink? How often? Do you drink together? What do they think about this?)*

**Is there anything else you’d like to say about alcohol before we move on?**

**Exercise**

**Can you tell me a bit about what sort of physical activity you do at the moment?**

*(Tell me a bit about this. What do you do? Where? How often? With whom? How do you feel this helps you?)*

**Do you exercise together with your partner?**

*(If yes, how often? If no, Why not? What do they think about this? Do you think exercising with your partner would/does help? How so?)*

**Have you made any changes to the amount of exercise you do since diagnosis?**

*(If yes, what changes? Why was this? Did you receive advice from a healthcare professional? Did you receive support from anyone? Did your partner make these changes? Were they aware you were making these changes? How did they feel about that?*

*If no, have you ever tried to make any changes in the past? What were these?)*

**Is there anything else you would like to say about exercise before we move on?**

**Intervention**

**We are exploring the idea of providing health advice to couples together, so that they can both make changes to eat more healthily and be more active.**

**Do you think this is something that you and your partner may have been interested in?**

*(why?)*

**How do you think this would work best?**

*(Advice from healthcare professional? Workshop together or with others? Through an app? Goal setting together?)*

**Is there anything else you can think of that would be helpful?**

**Conclusion**

**Is there something else that you would like to add about the role of your partner and health before we finish?**

**Or something else that feels important to discuss?**

**Do you have any questions for me?**

**Thank you for your time and participation.**

**General Prompts**

***Are you ok to talk to me a little about your original cancer diagnosis? What were your first thoughts after diagnosis? How did your husband/wife feel? How did you cope?***
